# Activation of CXCL-8 Transcription by Hepatitis E Virus ORF-1 via AP-1

**DOI:** 10.1155/2015/495370

**Published:** 2015-05-05

**Authors:** Zhubing Li, Lu Chen, Qiang Liu

**Affiliations:** ^1^VIDO-InterVac, Vaccinology and Immunotherapeutics Program, University of Saskatchewan, Saskatoon, SK, Canada S7N 5E3; ^2^VIDO-InterVac, University of Saskatchewan, Saskatoon, SK, Canada S7N 5E3; ^3^Henan Agricultural University, 95 Wenhua Road, Zhengzhou, Henan 450002, China; ^4^VIDO-InterVac, Vaccinology and Immunotherapeutics Program, Veterinary Microbiology, University of Saskatchewan, Saskatoon, SK, Canada S7N 5E3

## Abstract

Hepatitis E virus (HEV) is a small nonenveloped single-stranded positive-sense RNA virus and is one of the major causes for acute hepatitis worldwide. CXCL-8 is a small multifunctional proinflammatory chemokine. It was reported recently that HEV infection significantly upregulates CXCL-8 gene expression. In this study, we investigated the mechanism of HEV-induced CXCL-8 transcriptional activation. Using CXCL-8 promoter reporters of different lengths ranging from −1400 to −173, we showed that −173 promoter has the highest promoter activity in the presence of HEV genomic RNA, indicating that the −173 promoter contains sequences responsible for CXCL-8 activation by HEV. Ectopic expression of the ORF-1 protein can upregulate the −173 CXCL-8 promoter activity. In contrast, expression of the ORF-2 protein suppresses the CXCL-8 promoter activity and expression of the ORF-3 protein has no effect on the CXCL-8 promoter activity. We further showed that AP-1 is required for CXCL-8 activation because neither HEV genomic RNA nor the ORF-1 protein can upregulate the −173 CXCL-8 promoter in the absence of the AP-1 binding sequence. Taken together, our results showed that HEV and HEV ORF-1 protein activate the CXCL-8 promoter via AP-1. This novel function of HEV ORF-1 protein should contribute to our understanding of HEV-host interactions and HEV-associated pathogenesis.

## 1. Introduction

Hepatitis E virus (HEV), first identified in 1983, is a small nonenveloped single-stranded positive-sense RNA virus. It belongs to the genus* Hepevirus* in the Hepeviridae family and is classified into four genotypes [1, 2]. According to WHO, about two billion people are estimated to be infected with HEV with 14 million people showing clinical symptoms [3, 4]. HEV mainly causes self-limiting acute hepatitis, but the mortality rate is as high as 15–25% among pregnant women. Chronic hepatitis develops exclusively in immunocompromised patients infected with genotype 3 or 4 HEV [5–7].

HEV genome is approximately 7.2 kb and consists of three partially overlapping open reading frames (ORFs) flanked by short 5′ and 3′ untranslated regions [8, 9]. ORF-1 encodes a nonstructural polyprotein that can be cleaved into methyltransferase, papain-like cysteine protease, RNA helicase, and RNA-dependent RNA polymerase, which are essential for virus replication. ORF-2 encodes the viral capsid protein which contains neutralizing epitopes and ORF-3 encodes a small phosphoprotein that functions in virion morphogenesis, release, and pathogenesis [10–12].

CXCL-8, also known as interleukin-8, is a small multifunctional proinflammatory chemokine in the CXC chemokine family, which plays an important role in immune defence processes [13–15]. CXCL-8 is secreted at the inflammatory sites by different cells, such as neutrophils, macrophages, monocytes, and endothelial cells. CXCL-8 primarily targets the polymorphonuclear leukocytes which act as the first line of immune defence [16, 17]. Furthermore, CXCL-8 can also induce tumorigenesis, metastasis, and angiogenesis [17, 18]. CXCL-8 gene transcription can be regulated by NF-*κ*B and activator protein-1 (AP-1) [19].

Since it was recently shown that HEV infection significantly upregulates CXCL-8 gene expression [20], we investigated how HEV mediates CXCL-8 transcriptional activation. We showed that HEV genomic RNA and the ORF-1 protein upregulated CXCL-8 promoter-mediated transcription via AP-1.

## 2. Material and Methods

### 2.1. Plasmids

A cell culture adapted HEV genotype 3 Kernow C1 strain cDNA clone was received from Dr. Emerson [21]. The coding sequences of ORF-1, ORF-2, and ORF-3 were cloned into a pEF 3xFlag vector. Human CXCL-8 promoter-luciferase reporters of different lengths, −1400, −500, −230, −193, and −173, were received from Dr. Buendia [22]. Mutant −173 CXCL-8 promoters with mutations for the AP-1 binding sequence (^−126^TGACTCA^−120^ to ^−126^TatCTCA^−120^, mAP-1) [23] or the negative regulatory* cis*-element sequence (NRE) (^−76^ATTTCCTCTGA^−66^ to ^−76^ATTTCCcCcGA^−66^, mNRE) [24] were generated by site-directed mutagenesis. The AP-1-luciferase reporter was generated by inserting seven tandem repeats of the AP-1 binding sequence into the pGL4.27 vector (Promega). All plasmids were verified by DNA sequencing.

### 2.2. *In Vitro* Transcription

HEV genomic RNA was produced by* in vitro* transcription from linearized HEV cDNA using the mMESSAGE mMACHINE T7 kit (Ambion).

### 2.3. Cell Lines and Transfection

Human hepatoma HuH-7 and human embryonic kidney HEK293T cells were cultured in Dulbecco's Modified Eagle Medium (DMEM, Sigma-Aldrich) with 10% (v/v) fetal bovine serum (FBS, Life Technologies) at 37°C and 5% CO_2_. For luciferase assays, cells in 24-well plates were cotransfected with 1 *µ*g of HEV RNA and 1 *µ*g of luciferase reporter DNA using the jetPEI reagent (Polyplus), or with 0.5 *µ*g of plasmid DNA expressing HEV ORF proteins and 0.5 *µ*g of luciferase reporter DNA using the calcium phosphate precipitation method [25]. In the immunoprecipitation experiments, cells in 6-well plates were transfected with 2 *µ*g of plasmid DNA expressing HEV ORF proteins.

### 2.4. Luciferase Assay

Cells were lysed in Passive Lysis Buffer (Promega) and the luciferase activity was measured using a Luciferase Assay System (Promega). Luciferase activity was normalized to the protein concentration quantified using the Bradford assay reagent (Bio-Rad).

### 2.5. Immunoprecipitation, Western Blotting, and Antibodies

Cell lysates in RIPA buffer were immunoprecipitated with an anti-Flag antibody (Sigma-Aldrich) using the SureBeads magnetic beads system (Bio-Rad). Precipitated proteins were subjected to SDS-PAGE and transferred to nitrocellulose membranes. The blots were blocked in 5% skim milk in PBS for 1 hour and incubated with the anti-Flag antibody overnight at 4°C. After washing with PBS, the blots were incubated with an infrared dye-labeled secondary antibody (Li-Cor Biosciences) for 1 hour at room temperature and then washed again. The blot was scanned using Odyssey Infrared Imaging System (Li-Cor Biosciences).

### 2.6. Statistical Analysis

All experiments were performed three times in triplicate and differences between samples were assessed by Student's* t*-test. A* p* value less than 0.05 was considered statistically significant.

## 3. Results

### 3.1. Determination of CXCL-8 Promoter Activities in the Presence of HEV Genomic RNA

**Figure 1:**
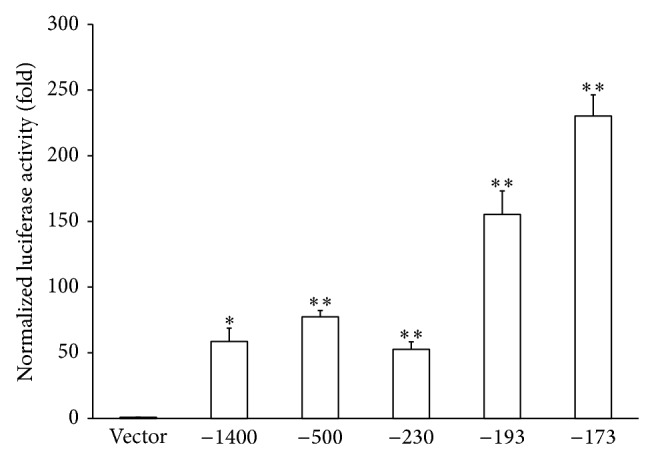
CXCL-8 promoter activities in the presence of HEV genomic RNA. HuH-7 cells were cotransfected with HEV genomic RNA and CXCL-8 promoter-luciferase reporters of different lengths or a luciferase reporter vector without the CXC-8 promoter. Luciferase assay was performed 48 hours after transfection. Luciferase activity was normalized against the protein concentration of the same samples. ∗:* p *≤ 0.05; ∗∗:* p *≤ 0.01 by Student's *t*-test.

A recent study showed that HEV infection can induce inflammatory cytokines and chemokines, including CXCL-8 [20]. To determine the minimum region of CXCL-8 promoter required for HEV enhanced CXCL-8 transcription, HuH-7 cells were cotransfected with HEV genomic RNA and CXCL-8 promoter-luciferase reporters of different lengths. The HEV genomic RNA can replicate and generate infectious HEV in cell culture [21]. Luciferase activity, as an indicator of CXCL-8 promoter activity, was measured 48 hours after transfection. As shown in Figure 1, we observed substantial luciferase activities when transcribed from the −1400, −500, −230, −193, and −173 CXCL-8 promoters. The −173 bp promoter displayed the highest promoter activity, suggesting that the −173 promoter contains the sequences necessary for CXCL-8 promoter activation by HEV.

### 3.2. CXCL-8 Promoter Activation by HEV ORF-1 via AP-1

**Figure 2:**
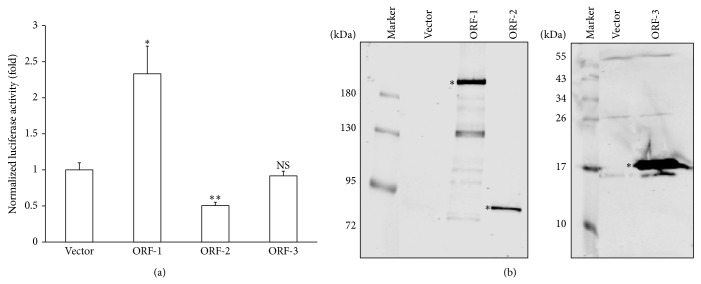
CXCL-8 transcriptional activation by HEV ORF-1. (a) HuH-7 cells were cotransfected with plasmid vector, or plasmids expressing HEV ORF-1, ORF-2, or ORF-3 proteins and the −173 CXCL-8 promoter-luciferase reporter. Luciferase assay was performed 48 hours after transfection. Luciferase activity was normalized against the protein concentration of the same samples. ∗:* p *≤ 0.05, ∗∗:* p *≤ 0.01, and NS: not significant by Student's* t*-test. (b) The expression of HEV ORF-1, ORF-2, or ORF-3 proteins with a N-terminal Flag-tag after transfection into HEK293 cells was analyzed by immunoprecipitation followed by Western blotting with an anti-Flag antibody. The location of HEV proteins was indicated by ∗ in the blot.

HEV encodes three proteins. To determine which HEV protein is responsible for activating the CXCL-8 promoter, we cotransfected HuH-7 cells with plasmids expressing the ORF-1, ORF-2, or ORF-3 proteins, or the vector control, together with the −173 CXCL-8 promoter reporter. As shown in Figure 2(a), the expression of the ORF-1 protein significantly increased CXCL-8 promoter activity in comparison to vector control, whereas the ORF-2 protein inhibited and the ORF-3 protein had no effect on CXCL-8 promoter activity. The expression of HEV proteins was confirmed by immunoprecipitation followed by Western blotting (Figure 2(b)). These results demonstrate that HEV ORF-1 protein can activate the CXCL-8 promoter.

**Figure 3:**
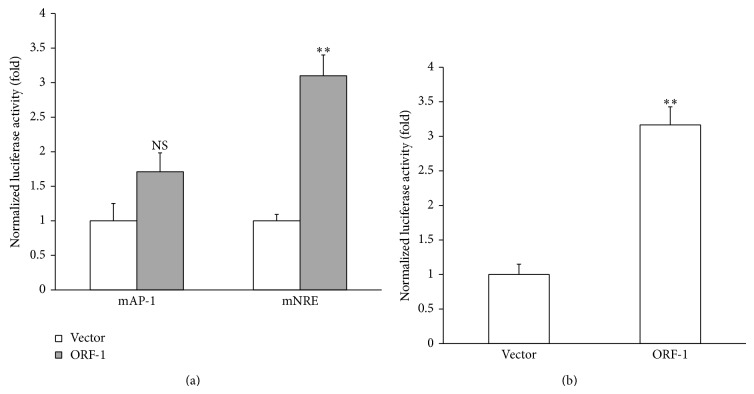
AP-1 is involved in CXCL-8 activation by HEV ORF-1. HuH-7 cells were cotransfected with plasmid vector or a plasmid expressing HEV ORF-1 protein, together with −173 mAP-1 or mNRE CXCL-8 promoter-luciferase reporters (a) or a luciferase reporter with seven repeats of the AP-1 binding sequences (b). Luciferase assay was performed 48 hours after transfection. Luciferase activity was normalized against the protein concentration of the same samples. ∗∗:* p *≤ 0.01; NS: not significant by Student's* t*-test.

The −173 CXCL-8 promoter contains the binding sequence for AP-1 [23]. To investigate the role of AP-1, the AP-1 binding sequence was removed from the CXCL-8 promoter by mutagenesis. As shown in Figure 3(a), the ORF-1 protein could no longer activate the mAP-1 CXCL-8 promoter. As a control, we used another mutant CXCL-8 promoter with the NRE sequence removed. HEV ORF-1 protein could activate the mNRE CXCL-8 promoter to a comparable level as to the wild-type CXCL-8 promoter (Figures 3(a) and 2(a)). These results demonstrate that AP-1 plays a role in CXCL-8 promoter activation by HEV ORF-1 protein.

### 3.3. AP-1 Upregulation by HEV ORF-1

Since the AP-1 binding sequence within the CXCL-8 promoter was shown to be required for activation by HEV ORF-1 protein, we hypothesized that HEV ORF-1 protein can directly increase transcription mediated by AP-1. We therefore cotransfected HuH-7 cells with a HEV ORF-1 protein expressing plasmid and a luciferase reporter plasmid with seven tandem repeats of the AP-1 binding sequence.  Figure 3(b) shows that the expression of HEV ORF-1 protein resulted in greater than threefold increase in the luciferase activity expressed downstream from the tandem AP-1 motifs when compared with basal promoter control. These results suggest that HEV ORF-1 protein can indeed upregulate AP-1-mediated transcription.

### 3.4. CXCL-8 Promoter Activation by HEV Genomic RNA Requires AP-1

**Figure 4:**
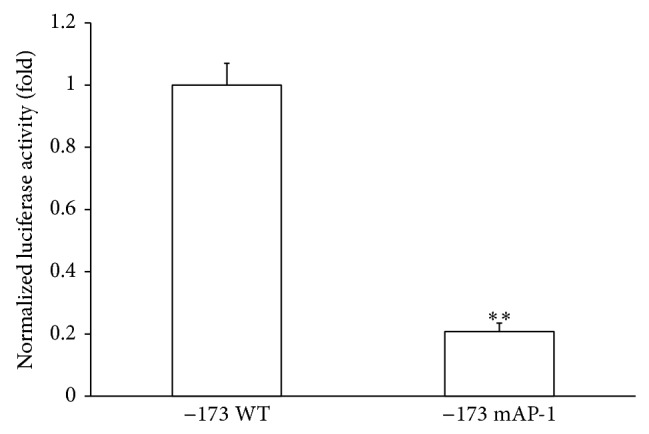
AP-1 is required for CXCL-8 activation by HEV genomic RNA. HuH-7 cells were cotransfected with HEV genomic RNA, together with the wild-type or mAP-1 −173 CXCL-8 promoter-luciferase reporters. Luciferase assay was performed 48 hours after transfection. Luciferase activity was normalized against the protein concentration of the same samples. ∗∗:* p *≤ 0.01 by Student's* t*-test.

Although we showed that AP-1 is involved in CXCL-8 promoter activation by HEV ORF-1, it was important to demonstrate that transcriptional activation of the CXCL-8 promoter by HEV also requires AP-1. To address this question, we cotransfected HuH-7 cells with the HEV genomic RNA, together with wild-type or mAP-1 −173 CXCL-8 promoter-luciferase reporters. Results showed that elimination of the AP-1 binding sequence resulted in a significantly lower luciferase activity in comparison to the wild-type promoter (Figure 4). This finding demonstrates the importance of AP-1 in CXCL-8 promoter activation by HEV.

## 4. Discussion

Inflammatory cytokines and chemokines play a critical role in the pathology associated with viral infections. Although elevated inflammatory cytokine/chemokine levels are detected in HEV-associated liver failure patients [26], there is very limited information on how HEV regulates these genes at the molecular level. Devhare et al. showed upregulation of several inflammatory cytokine/chemokine genes and downregulation of interferons after HEV infection in a cell culture model [20]. Consistently, we showed that transfection of a HEV genomic RNA is associated with substantial CXCL-8 transcription in a promoter-luciferase reporter assay. We further demonstrated that the ORF-1 protein, but not the ORF-2 or ORF-3 proteins, can activate CXCL-8 promoter. In addition, we identified the requirement of AP-1 in CXCL-8 transcription activation by both HEV genomic RNA and the ORF-1 protein.

Amongst the CXCL-8 promoters of different lengths ranging from −1400 to −173, the −193 and −173 promoters exhibit higher degree of activities in the presence of HEV RNA (Figure 1). The mechanisms of this differential activation are not fully understood. The activity of a promoter is determined by the interplay of positive and negative factors involved. The binding motifs for multiple transcriptional factors, such as IRF-1, HNF-1, and GR, are located between the −1400 and −173 promoter region [22]. Therefore, it is possible that multiple transcription factors are involved in regulating CXCL-8 transcription by HEV. Furthermore, a few mechanisms, such as histone deacetylation and OCT-1 binding, have been shown to inhibit the CXCL-8 promoter activity [27]. Whether these mechanisms play a role in CXCL-8 promoter regulation by HEV requires further investigation.

The ORF-1 protein, essential for HEV RNA replication, consists of a few domains which can function as methyltransferase, cysteine protease, RNA helicase, and RNA-dependent RNA polymerase [6]. It would be interesting to determine which domains of ORF-1 are responsible for activating CXCL-8 transcription. The cysteine protease domain and the adjacent X domain have been shown to inhibit interferon transcription [28]. Thus, the ORF-1 protein can both upregulate inflammatory chemokine expression and downregulate interferon expression after HEV infection.

The capsid ORF-2 protein, when expressed as an intracellular protein, was found to inhibit CXCL-8 transcription (Figure 2(a)). Previous studies have shown that the UV-inactivated HEV virus, but not soluble ORF-2 protein, induces inflammatory cytokines and chemokines [20]. This indicates that the ORF-2 protein has different functional properties as a component of the viral capsid compared with an intracellular protein. Further studies are required to study the functions of the ORF-2 protein.

## 5. Conclusions

We showed that HEV genomic RNA and the ORF-1 protein significantly enhance CXCL-8 promoter activity which requires the transcription factor AP-1. This newly identified function of the ORF-1 protein should help understand the molecular mechanisms of HEV-host interactions and HEV-associated pathogenesis.
